# Evaluation of retrograde bacterial migration in a prototype urinary drainage system with a silicone oil barrier

**DOI:** 10.1016/j.infpip.2026.100585

**Published:** 2026-08-25

**Authors:** Martin Slettengren, Jan van der Linden

**Affiliations:** a Department of Molecular Medicine and Surgery, Karolinska Institutet, Stockholm, Sweden; b Department of Physiology and Pharmacology, Division for Anesthesiology and Intensive Care, Karolinska Institutet, Stockholm, Sweden; c Department of Anesthesiology, Karolinska University Hospital, Stockholm, Sweden

**Keywords:** Catheter-associated urinary tract infection (CAUTI), Urinary drainage system, Retrograde, bacterial migration, *Proteus mirabilis*, Infection prevention, Physicochemical barrier

## Abstract

**Background:**

Retrograde contamination of urinary drainage systems is a recognised pathway contributing to catheter-associated urinary tract infection. We evaluated retrograde bacterial migration in a prototype urinary drainage system incorporating a silicone oil barrier during a 14-day in-vitro challenge.

**Methods:**

An automated in-vitro model (*N* = 7) simulated clinical conditions, including continuous flow (29 mL/h), physiological temperature (37 °C), hourly chamber emptying, daily bag emptying, and repeated 90° tilting to mimic patient transport and repositioning. Collection bags were inoculated with *Proteus mirabilis* CCUG 26767ᵀ. After 14 days, six predefined locations were analysed for microbiological culture and field-emission scanning electron microscopy (FE-SEM).

**Results:**

*P. mirabilis* was detected in all collection bags (7/7) and in one of seven bag inlets (1/7). No bacterial growth was observed in the chamber, drainage tubing, or catheter connector (0/7), indicating the absence of detectable retrograde migration to proximal components. FE-SEM confirmed biofilm formation confined to distal components, with no bacterial attachment detected proximally.

**Conclusions:**

In this in-vitro study, no detectable retrograde migration of *P. mirabilis* was observed in the prototype system during a 14-day dynamic challenge. Further studies using larger sample sizes, additional uropathogens, concurrent control systems, and clinical evaluation are warranted to determine the contribution of the barrier mechanism and its potential clinical applicability.

## Background

Catheter-associated urinary tract infections (CAUTIs) remain among the most common healthcare-associated infections and contribute substantially to patient morbidity and healthcare costs [[1], [2], [3]]. The risk of bacteriuria increases with the duration of catheterisation, reflecting progressive bacterial colonisation of the catheter and urinary drainage system. Retrograde contamination of the drainage system has been recognised as one pathway contributing to catheter colonisation and subsequent infection [1,[4], [5], [6]].

*Proteus mirabilis* is a particularly suitable organism for evaluating retrograde bacterial migration because of its pronounced swarming motility and urease activity, which promote crystalline biofilm formation, catheter encrustation, and urinary tract obstruction [1,4,5].

Although urinary drainage systems incorporate anti-reflux valves, retrograde bacterial migration from the collection bag may still occur during routine patient handling, repositioning, and transport [6]. Rasmussen *et al.* developed an experimental model demonstrating that retrograde migration depends on drainage system design [7]. Subsequently, silicone oil was shown to reduce microbial adhesion and biofilm formation on medical device surfaces in multiple clinically relevant bacterial and fungal species [8].

Building on these observations, we evaluated retrograde bacterial migration in a prototype urinary drainage system incorporating a silicone oil barrier using a dynamic in-vitro model based on that described by Rasmussen *et al.* [7].

## Methods

### Test system and barrier integration

In this experimental study, 7 prototypes of the UnoMeter™ Safeti™ Max (Item no. 120075, lot no. HD010001) were evaluated for retrograde bacterial migration of *P. mirabilis* over 14 days. The prototypes were modified versions of the UnoMeter™ Safeti™ Plus and included a silicone oil capsule mounted on the measurement chamber lid. The capsule releases a medium-viscosity, medical-grade polydimethylsiloxane silicone oil (Silbione® 70047 V350; Elkem, Oslo, Norway; viscosity: 350 mm^2^/s at 25 °C) [8,9], which forms a hydrophobic layer on the polypropylene chamber surfaces. This layer was intended to disrupt the continuous liquid interface required for swarming motility of *P. mirabilis*. Barrier integrity was monitored visually, and no macroscopic disruption or depletion of the silicone oil layer was observed during the 14-day experiment. Before installation, each chamber tap was manually cycled 5 times to ensure mechanical function and initial oil distribution.

### In-vitro model and clinical simulation

A modified in-vitro model based on the experimental system described by Rasmussen *et al.* [7] was used to simulate clinically relevant drainage conditions. The drainage systems were mounted in a custom fixture providing automated control of flow and mechanical movement throughout the 14-day experiment. The automated protocol included hourly opening of the chamber tap to transfer medium to the collection bag, simulating regular bladder emptying. To simulate patient repositioning and transport, the fixture performed automated 90° tilting twice daily for 10 min.

### Experimental conditions and inoculation

The growth medium consisted of tryptic soy broth (TSB) diluted 1:9 with physiological saline, consistent with the experimental model described by Rasmussen *et al.* [7]. This medium was selected to approximate nutrient-limited conditions while maintaining bacterial viability during prolonged continuous-flow experiments. Synthetic urine was not used because the objective was to maintain stable bacterial growth rather than reproduce the complete biochemical composition of urine.

The medium was maintained at 37 °C and delivered at a constant flow rate of 29 mL/h via a peristaltic pump (Longer Pump® BT100-1L). The inoculum was prepared from colonies subcultured from frozen stock cultures on blood agar plates. Two to three colonies were transferred to 10 mL of TSB and incubated aerobically at 37 °C for 18–24 h. The culture was then adjusted to approximately 10^6^ colony-forming units/mL based on optical density measurements, and the concentration was verified by quantitative plate count on UriSelect agar before inoculation. Each collection bag was inoculated with 1 mL of the suspension after 100 mL of sterile medium had accumulated. Each drainage system was operated independently as a separate experimental replicate throughout the 14-day challenge.

### Biofilm and structural analysis

After the 14-day challenge, the drainage systems were disconnected for sampling. Biofilm formation and surface colonisation were analysed by field-emission scanning electron microscopy (FE-SEM; Zeiss Supra 40VP). Samples were prepared using a GATAN Model 682 PECS sputter coater for high-resolution visualisation of bacterial attachment and extracellular matrix structures.

### Sampling protocol and locations

**Figure 1:**
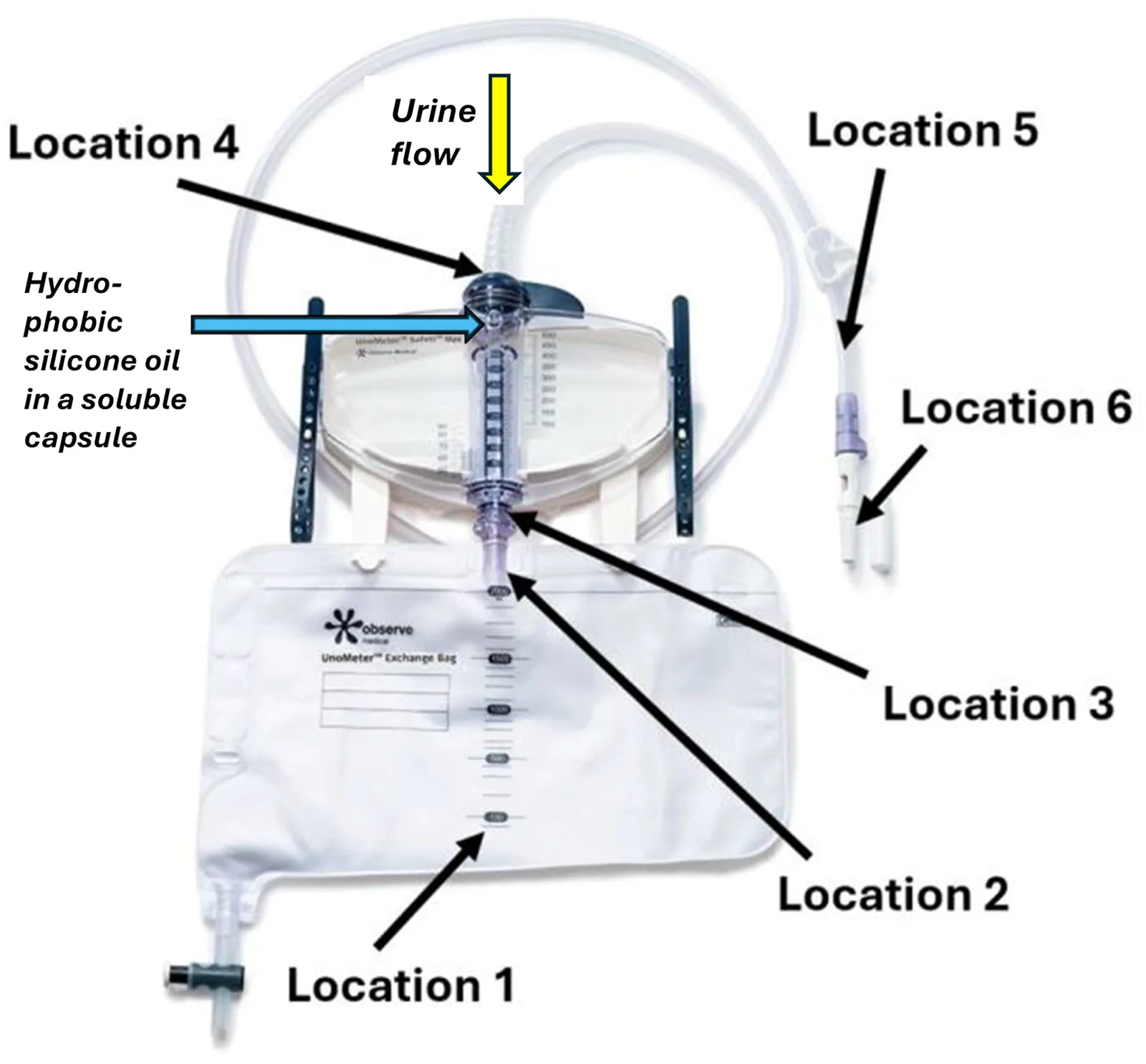
Schematic of the prototype urinary drainage system and sampling locations. The drainage system incorporated a silicone oil capsule mounted on the measurement chamber, where released silicone oil formed a hydrophobic barrier at the chamber interface. Six predefined sampling locations were sampled and analysed after the 14-day challenge to assess retrograde migration of *Proteus mirabilis*.

After the 14-day challenge, six predefined locations were sampled under sterile conditions to assess retrograde migration of *P. mirabilis* (Figure 1): the collection bag, bag inlet, chamber outlet, drainage tubing (two locations), and connector cone. Samples were collected using sterile saline-moistened swabs.

Location 1 (collection bag): 5 × 5-cm area centred on the 100-mL graduation mark.

Location 2 (bag inlet): internal surface of the inlet tube.

Location 3 (chamber outlet): outlet between the measurement chamber and collection bag.

Location 4 (distal drainage tubing): catheter-side tubing immediately after the chamber.

Location 5 (proximal drainage tubing): tubing 5 cm proximal to the sampling port.

Location 6 (connector cone): internal circumference of the connector cone.

Swabs were cultured on UriSelect agar for 24 h at 37 °C. *P. mirabilis* was identified by characteristic colony morphology and prior confirmation of the inoculum strain. Corresponding locations were prepared for the FE-SEM analysis. Detailed materials are listed in Supplementary Table A1. All microbiological and ultrastructural analyses were performed independently at the RISE Research Institutes of Sweden (Gothenburg, Sweden).

### Statistical analysis

The primary outcome was the presence or absence of bacterial colonisation at each predefined sampling location after 14 days. The study was designed to determine whether retrograde bacterial migration occurred beyond the collection bag rather than to quantify bacterial burden at each sampling location. Given the small sample size and binary outcome, results are presented descriptively. Fisher's exact test was used to compare distal and proximal colonisation patterns.

## Results

### Microbiological analysis

After 14 days, *P. mirabilis* colonisation was detected in all collection bags (7/7; location 1) and in one of seven bag inlets (1/7; location 2, Table I). No bacterial growth was detected in the chamber outlet, drainage tubing, or catheter connector (0/7; locations 3–6). Colonisation differed significantly between distal (locations 1–2) and proximal (locations 3–6) drainage components (Fisher's exact test, *P* < 0.001).

**Table I:** Culture and FE-SEM findings at six sampling locations after a 14-day *Proteus mirabilis* challenge (*N* = 7)

Sampling location	Description	Culture (*N*/7)	Detection rate (%)	Biofilm detection by FE-SEM
**Distal components**				
Location 1	Collection bag	7/7	100%	Detected
Location 2	Bag inlet	1/7	14%	Detected
**Proximal drainage components**				
Location 3	Chamber outlet	0/7	0%	Not detected
Location 4	Drainage tubing (catheter side)	0/7	0%	Not detected
Location 5	Drainage tubing (5 cm from sampling port)	0/7	0%	Not detected
Location 6	Catheter connector cone	0/7	0%	Not detected

FE-SEM, field-emission scanning electron microscopy.

Colonisation differed significantly between distal (locations 1–2) and proximal (locations 3–6) components (Fisher’s exact test, *P* < 0.001).

### Ultrastructural analysis (FE-SEM)

**Figure 2:**
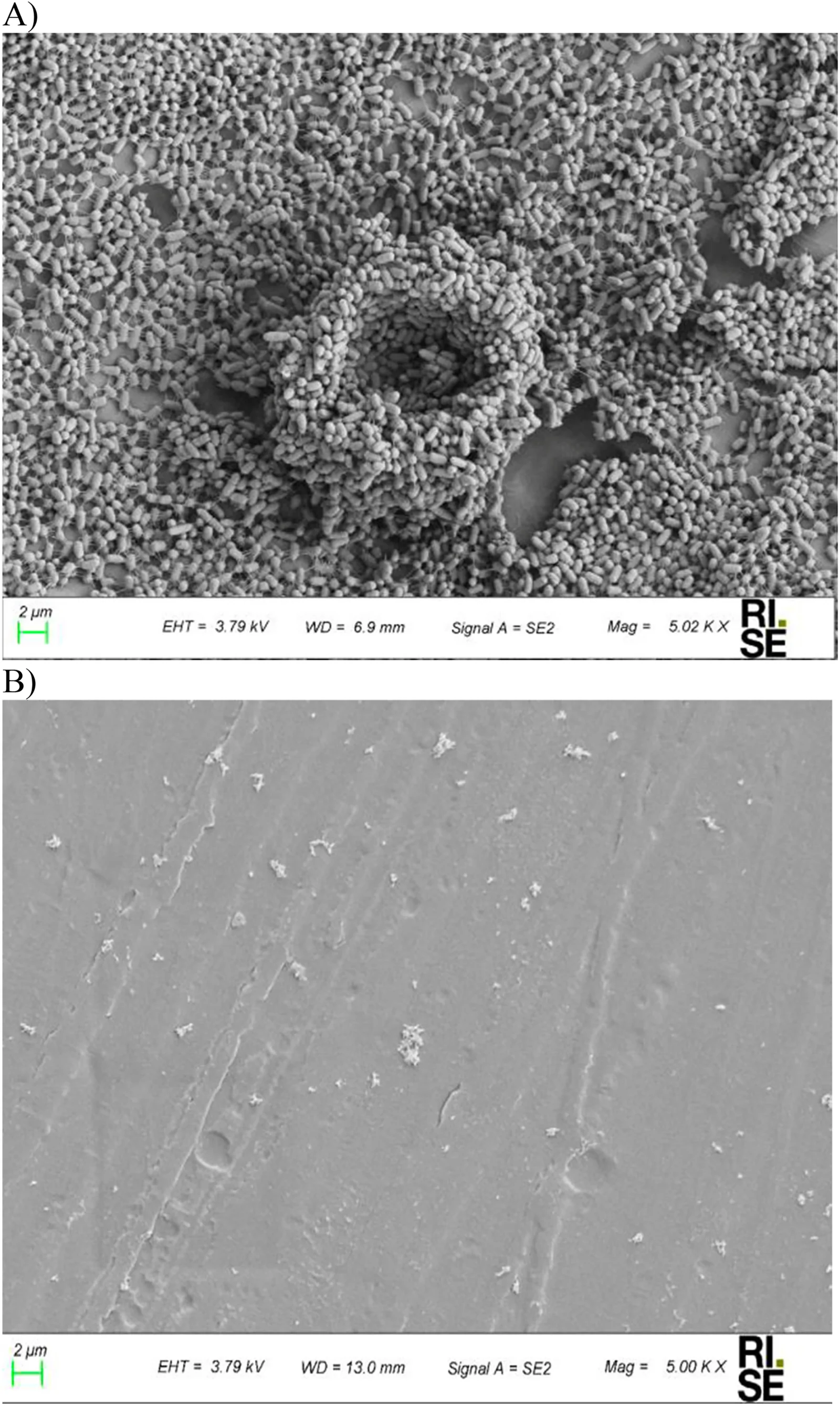
FE-SEM micrographs of drainage system surfaces after a 14-day *Proteus mirabilis* challenge. (A) Collection bag (location 1) showing mature biofilm with extracellular matrix. (B) Connector cone (location 6) showing no detectable bacterial attachment or biofilm, consistent with the absence of retrograde colonisation in proximal drainage components. Scale bars = 2 μm. FE-SEM, field-emission scanning electron microscopy.

FE-SEM imaging corroborated the microbiological findings (Figure 2). Dense biofilm with extracellular matrix structures was observed at the collection bag and bag inlet (locations 1–2), whereas no bacterial attachment or biofilm was detected in the proximal drainage components (locations 3–6). These findings were consistent with the microbiological culture results.

## Discussion

In this in-vitro study, no detectable retrograde migration of *P. mirabilis* was observed in a prototype urinary drainage system with a silicone oil physicochemical barrier during a 14-day challenge. Despite continuous bacterial contamination of the collection bag and repeated mechanical stress, no bacterial growth was detected in the proximal drainage components. These findings suggest that limiting retrograde bacterial migration within the drainage pathway may represent a potential infection prevention strategy. The findings are consistent with the biological requirement for a continuous liquid film to support the swarming motility of *P. mirabilis*.

Rasmussen *et al.* demonstrated that retrograde bacterial migration depends on drainage system design and occurred in most urinary drainage systems despite the presence of anti-reflux valves [7]. In the present study, no detectable retrograde migration of the highly motile organism *P. mirabilis* was observed in a prototype urinary drainage system incorporating a silicone oil barrier during a 14-day dynamic challenge with repeated 90° tilting. A previous study by our group demonstrated that silicone oil reduces microbial adhesion and biofilm formation in several clinically relevant bacterial and fungal species, including *Escherichia coli* (including Extended Spectrum Beta Lactamases (ESBL)-producing strains), *Klebsiella pneumoniae* (including multi-drug-resistant strains), *Pseudomonas aeruginosa, Enterococcus faecalis, Staphylococcus aureus*, and *Candida albicans* [8]. However, because the present study did not include a concurrent control drainage system, the independent contribution of the silicone oil barrier cannot be determined. Nevertheless, the present findings extend previous experimental findings by demonstrating the absence of detectable retrograde migration under dynamic test conditions.

Strengths of the study include the automated dynamic in-vitro model and confirmation of microbiological findings by FE-SEM. The principal limitation is the absence of a concurrent control drainage system without the silicone oil barrier. Therefore, the independent contributions of the silicone oil barrier and drainage system design cannot be determined. In addition, only a single bacterial species was evaluated in the present urinary drainage model. Furthermore, the study was designed to assess the presence or absence of retrograde migration rather than quantitative bacterial burden at each sampling location. Although a previous study by our group demonstrated reduced microbial adhesion and biofilm formation with silicone oil across multiple clinically relevant bacterial and fungal species [8], further studies using additional uropathogens, concurrent control systems, and clinical evaluation are warranted.

Although extraluminal ascent along the catheter is considered an important route in CAUTI pathogenesis, intraluminal contamination of urinary drainage systems has also been recognised as a contributing pathway [10]. Strategies that limit ascending contamination may therefore be particularly relevant during short-term catheterisation, when bacteriuria develops progressively.

In conclusion, no detectable retrograde migration of *P. mirabilis* was observed in this prototype urinary drainage system during a 14-day in-vitro challenge. These findings support further investigation of silicone oil barrier technology as a potential complementary strategy to reduce retrograde bacterial migration and limit ascending contamination within urinary drainage systems. Because this was a small in-vitro study without a concurrent control system, the findings should be interpreted cautiously and do not rule out bacterial migration below the detection limit. Further studies, including larger experimental studies with additional uropathogens and concurrent control systems, as well as clinical evaluation, are warranted to determine whether this approach can contribute to the prevention of catheter-associated urinary tract infections.

## CRediT authorship contribution statement

**Martin Slettengren:** Writing – original draft, Project administration, Methodology, Investigation, Formal analysis, Data curation, Conceptualization. **Jan van der Linden:** Writing – review & editing, Supervision, Resources, Methodology, Funding acquisition, Conceptualization.

## Ethical approval

Not required.

## Funding sources

Observe Medical AB (Mölndal, Sweden) funded the study; however, the experimental work and microbiological analyses were conducted independently at the RISE Research Institutes of Sweden (Gothenburg, Sweden).

## Conflict of interest statement

This study was funded by Observe Medical AB (Mölndal, Sweden). The experimental laboratory work was conducted independently by the RISE Research Institutes of Sweden (Gothenburg, Sweden). Jan van der Linden serves as a consultant for Observe Medical AB. The authors declare no other competing interests.
