# Supplementary material for: Evaluation of retrograde bacterial migration in a prototype urinary drainage system with a silicone oil barrier
Source: Infect Prev Pract. 2026 Aug 25;8(4):100585. doi: 10.1016/j.infpip.2026.100585 (PMC13598591; doi:10.1016/j.infpip.2026.100585)
Supplement: Supplementary file 1 [file mmc1.docx]

**Supplementary Table A1.** Materials, reagents, and equipment used in the in vitro study.

| **Material / reagent** | **Manufacturer** | **Catalog number** | **Batch / lot number** |
| --- | --- | --- | --- |
| **Test item** |  |  |  |
| Unometer™ Safeti™ Max prototype | Observe Medical AB | 120075 | HD010001 |
| **Test organism** |  |  |  |
| *Proteus mirabilis* CCUG 26767ᵀ (type strain) | CCUG | 26767ᵀ | – |
| **Culture media and solutions** |  |  |  |
| Tryptic Soy Broth (TSB) | Oxoid | CM0129B | 6349817 |
| Sodium chloride (99.5%) | Thermo Fisher Scientific | 207790050 | A0460165 |
| Blood agar plates | Sahlgrenska University Hospital | – | 310 |
| UriSelect agar plates | Bio-Rad | 64694 | 400 |
| **SEM fixatives** |  |  |  |
| Glutaraldehyde solution (50%) | Sigma-Aldrich | 340855 | – |
| Osmium tetroxide (4%) | Thermo Fisher Scientific | 191185000 | – |
| Hexamethyldisilazane | Thermo Fisher Scientific | A15139.AE | – |
| **Consumables** |  |  |  |
| Sterile swabs | Copan | 159C | 187A45 |
| Disposable sterile scalpels | Swann-Morton | 0506 | 7112312 |
| Silicone tubing  (2 × 4 mm) | VWR | 228-0704 | 6867263 |
| Silicone tubing  (5 × 8 mm) | VWR | BRND143358 | 26A |
